# Acupuncture in the treatment of fatigue in Parkinson's disease: A pilot, randomized, controlled, study

**DOI:** 10.1002/brb3.897

**Published:** 2017-12-29

**Authors:** Keng H. Kong, Hwee L. Ng, Wei Li, Dora W. Ng, Siang I. Tan, Kay Y. Tay, Wing L. Au, Louis C. S. Tan

**Affiliations:** ^1^ Complementary Integrative Medicine Tan Tock Seng Hospital Singapore City Singapore; ^2^ Neurology, National Neuroscience Institute Singapore City Singapore

**Keywords:** acupuncture, fatigue, Parkinson's disease, randomized controlled trial

## Abstract

**Introduction:**

Fatigue is a common and disabling problem in patients with Parkinson's disease (PD), and there is currently no satisfactory treatment. As acupuncture has been reported to be effective in fatigue related to other conditions, we sought to evaluate its efficacy in PD.

**Methods:**

This was a single center, randomized, sham‐controlled study. Forty PD patients with moderately severe fatigue were randomized to receive 5 weeks of biweekly real or sham acupuncture. The primary outcome was change on the General Fatigue score of the Multidimensional Fatigue Inventory (MFI‐GF) at 5 weeks. Secondary outcomes included MFI‐Total score, Unified Parkinson's Disease Rating Scale Motor score (UPDRS Motor), Parkinson's Disease Questionnaire‐39 (PDQ 39), Geriatrics Depression Scale (GDS), and Epworth Sleepiness Scale ESS). All outcome measures were evaluated at baseline, 5 and 9 weeks.

**Results:**

Both groups showed significant improvements in MFI‐GF and MFI‐Total scores at 5 and 9 weeks, but there were no significant between‐group differences. There were no improvements from baseline for PDQ 39, GDS, and ESS. Although improvements were noted for the UPDRS Motor score in the real acupuncture group, no between‐group difference could be demonstrated.

**Conclusions:**

Both real and sham acupuncture are equally effective in improving PD‐related fatigue, and it is likely that this is due to nonspecific or placebo effects.

## 1 INTRODUCTION

Fatigue is one of the commonest nonmotor symptoms in Parkinson's disease (PD) and has negative impact on quality of life (Barone et al., 2009; Friedman, Abrantes, & Sweet, 2011; Skorvanek et al., 2013). In one of the first studies on fatigue in PD, 15–33% of patients rated it as their most disabling symptom, and more than half rated fatigue among their three worst symptoms (Havlikova et al., 2008). There is currently no satisfactory treatment of PD‐related fatigue (Franssen, Winward, Collett, Wade, & Dawes, 2014).

Acupuncture has been shown to be effective in the treatment of fatigue related to other conditions, in particular, cancer‐related fatigue (Molassiotis et al., 2012; Sood, Barton, Bauer, & Loprinzi, 2007). Apart from a recent study (Kluger et al., 2016) its role in PD‐related fatigue has not been explored. The aim of this randomized, controlled pilot study was to evaluate the efficacy of a 5‐week course of acupuncture in the treatment of fatigue in PD.

## 2 PATIENTS AND METHODS

### 2.1 Study design

This was a randomized, patient and assessor‐blinded, controlled pilot study assessing the efficacy of acupuncture in patients with PD‐related fatigue.

### 2.2 Subjects and setting

Participants attending the Parkinson's Disease Clinic of National Neuroscience Institute‐Tan Tock Seng Hospital, Singapore who met the following inclusion and exclusion criteria were recruited. The inclusion criteria were (1) diagnosis of PD based on criteria developed by Gelb, Oliver, & Gilman (1999) which is adopted by the National Institute of Neurological Disorders and Stroke, US National Institute of Health, (2) age 21–85 years old, (3) presence of moderately severe fatigue as defined by a score of ≥10 on the General Fatigue domain of the Multidimensional Fatigue Inventory (Smets, Grasen, Bonke, & De Haes, 1995), and (4) no acupuncture treatment in the past 6 months.

The exclusion criteria were (1) significant cognitive, language or psychiatric illnesses which prevents the subject from understanding instructions and participating in the study, (2) needle phobia, (3) comorbidity with a bleeding disorder, (4) known anemia with hemoglobin level <10 g/dl, (5) known congestive cardiac failure and/or end‐stage renal disease, (6) female subjects of childbearing age, and (7) presence of symptomatic postural hypotension.

### 2.3 Screening and randomization

Opportunistic screening of patients attending the Parkinson's Disease Clinic of National Neuroscience Institute‐Tan Tock Seng Hospital, Singapore was conducted. After informed consent, eligible patients were randomized in a 1:1 fashion without stratification to real or sham acupuncture using permuted blocks. Allocation to treatment group was managed by an interactive web response system using computer‐generated lists organized by a statistician who is independent of the study. Only the two treating acupuncturists had access to the interactive web response system. All other study team members were blind to group assignment.

### 2.4 Intervention

Treatment consisted of twice‐weekly sessions at least 3 days apart for 5 weeks, giving a total of 10 sessions of acupuncture. Acupuncture was performed with the patient supine on a acupuncture table. The retractable noninvasive sham and invasive acupuncture needles developed by Jongbae Park (Park Sham Device, PSD) were used in this study (Park, White, Stevinson, Ernest, & James, 2002). Both real and sham needles have a fine needle body and copy handle and look exactly the same. However, the retractable needle has a retractable shaft and blunt tip. When pressed onto the skin, it telescopes into the handle and the blunt tip stays on the skin instead of penetrating it. The plastic tube with adhesive foot‐plate is placed on the skin to hold it in place. The real needle, on the other hand, has a normal sharp tip which allows it to pierce the skin. Both needles are 70 mm long. The Park Sham needle has been well validated as an inactive and credible placebo control in clinical acupuncture trials (Park, White et al., 2002).

For patients in the real acupuncture group, acupoints were needled to a depth of 0.5 to 1 inch, depending on patient's size and sensitivity in the following order: right PC 6, left PC 6, right LI 4, left LI 4, right ST 36, left ST 36, right SP 6, left SP 6, right KI 3, left KI 3, and CV 6. Thus, a total of 11 acupoints were needled at each session. No flicking or rotation of needles was allowed after insertion. The needles were retained in position for 20 minutes after which they were removed. Each session was based on a strict protocol, and conversation between acupuncturists and patients was kept to a minimum.

The acupoints were chosen based on Traditional Chinese Medicine theory and the protocol used by Molassiotis in cancer‐related fatigue (Molassiotis et al., 2012). No other complementary therapy was recommended during the course of the study documented. All intervention was performed by two licensed acupuncturists with at least 5 years of clinical experience.

### 2.5 Primary outcome measure

The primary outcome measure was the change in General Fatigue domain score of the Multidimensional Fatigue Inventory (MFI) from baseline at 5 weeks. The MFI is a 20‐item self‐reporting tool that measures five dimensions of fatigue: General Fatigue, Physical Fatigue, Reduced Activity, Reduced Motivation, and Mental Fatigue. Each subscale contains four items, which are scored on a five‐point Likert scale. Scores range from 4 (absence of fatigue) to 20 (maximum fatigue) for each subscale. Its reliability and structural validity in patients with idiopathic PD have been published (Elbers, van Wegen, Verhoef, & Kwakkel, 2012).

### 2.6 Secondary outcome measures

1. Motor function was quantified using the motor subsection of the Unified Parkinson's Disease Rating Scale (UPDRS; Fahn & Elton, 1987).
2. Parkinson's Disease Questionnaire‐39 (PDQ 39) to evaluate the quality of life (Jenkinson, Fitzpatrick, Peto, Greenhall, & Hyman, 1996). The questionnaire provides scores on eight dimensions: mobility, activities of daily living, emotions, stigma, social support, cognition, communication, and bodily discomfort.
3. Geriatric Depression Scale‐Short Form (GDS) for the evaluation of mood (Sheik & Yesavage, 1986).
4. Epworth Sleepiness Scale (ESS) for the evaluation of excessive daytime somnolence (Johns, 1991).

Information on socio‐demographic characteristics, disease duration, antiparkinsonian medications, and other treatment was also obtained. The levodopa equivalent daily dosage was counted using the formula published by Tomlinson et al. (2010) Participants were assessed at Week 0 (baseline), Week 5 (completion of intervention), and Week 9 (4 weeks after completion of intervention). At Week 5, participants were also questioned on their perceived group assignment (acupuncture, not sure, sham) to test blinding adequacy.

### 2.7 Statistical analysis

In a previous study of prevalence of fatigue in PD using the MFI, the mean General Fatigue score was 15.2 with a standard deviation of 3.0 (Skorvanek et al., 2013). Assuming a 20% reduction in MFI score in the acupuncture group compared to control, a two‐sided sample comparison of means requires 16 participants per group with significance level at 0.05 and power of 0.8. Hence, a total of 32 participants need to be recruited. Assuming a drop‐out rate of 20%, the number of participants needed will be 40. The effect size of 20% was chosen on the premise that anything less than this is not likely to be clinically significant. All analyses were conducted according to intention to treat principles. Descriptive statistics were computed for each of the analyzed variables. Within‐group changes between baseline and Week 5 were performed using paired t tests. Analysis of covariance (ANCOVA) modeling was used to examine the effect of group assignment on General Fatigue score and secondary outcomes. A last value carried forward approach was used as a sensitivity analysis for subjects with missing values between the two groups. All testing was two‐sided and results were deemed significant if *p* value <.05, using SPSS version 20.

This study was approved by the National Healthcare Group, Singapore Institutional Review Board. The trial was registered with ClincalTrials.gov (NCT02587754).

## 3 RESULTS

### 3.1 Baseline characteristics

**Figure 1:**
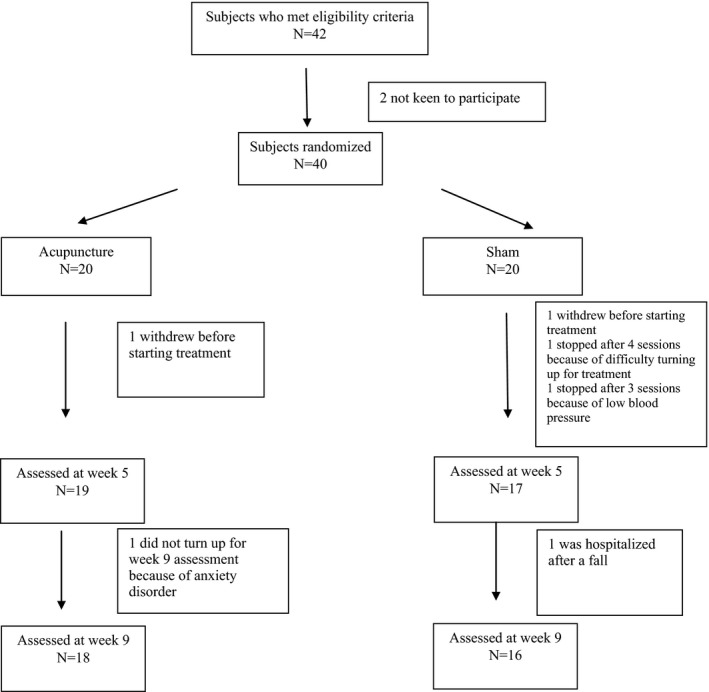
Study flowchart and CONSORT diagram

Between October 2015 and Nov 2016, 42 patients met eligibility criteria, but only 40 consented to the study (see Figure 1) Thirty‐six patients (90%) completed the assigned intervention at Week 5 and 34 (85%) at week 9. Table 1 shows the demographic and clinical characteristics of the study cohort. Of note is the finding that all patients were of Chinese ethnicity. There were no statistically significant differences between groups, except for duration of PD (longer in the acupuncture group) and PDQ 39 score (higher in the acupuncture group).

**Table 1:** Group demographics and characteristics

	Overall mean ± *SD* or *n* (%)	Acupuncture mean ± *SD* or *n* (%)	Sham mean ± *SD* or *n* (%)	*p* Value
Age (years)	64.6 ± 8.4	66.4 ± 6.5	62.9 ± 9.7	.19
Female	27 (67.5)	14 (70.0)	13 (65.0)	.73
Duration of PD (months)	68.8 ± 45.5	87.2 ± 53.2	50.1 ± 26.4	**.008**
Levodopa equivalent dosage	615.2 ± 347.9	637.8 ± 394.3	592.6 ± 303.1	.68
MFI – Total	59.3 ± 12.8	61.6 ± 15.1	56.6 ± 9.2	.23
MFI – General Fatigue	13.4 ± 2.8	13.4 ± 2.9	14.0 ± 2.7	.47
UPDRS Motor	25.4 ± 12.4	27.1 ± 13.7	23.8 ± 10.9	0.39
PDQ‐39	31.2 ± 22.6	38.4 ± 25.1	23.7 ± 17.5	**0.042**
ESS	7.1 ± 4.0	7.5 ± 3.8	6.7 ± 4.4	0.59
GDS	3.6 ± 2.9	4.0 ± 3.3	3.3 ± 2.5	0.44

*p* ‐ values that are in bold shows statistical significance.

### 3.2 Primary outcome measure

Both real and sham acupuncture groups demonstrated significant improvements in the MFI‐General Fatigue scores at Week 5 (change in MFI‐General Fatigue scores from baseline to Week 5: acupuncture, −2.7 ± 2.9; sham, −4.6 ± 2.8; see Table 2). However, this difference was not statistically significant between groups (ANCOVA, *p* = .09).

**Table 2:** Mean scores of outcome measures at baseline and on follow up and between‐group differences

	Acupuncture	*p* ValueChange from baseline	Sham	*p* ValueChange from baseline	*p* Value from ANCOVA
MFI – GENERAL FATIGUE					
Baseline	13.4 ± 2.9		14.0 ± 2.7		
Week 5	10.4 ± 3.3	.001	9.3 ± 2.9	<.001	.09
Week 9	10.6 ± 3.37	.006	10.5 ± 3.2	<.001	.60
MFI – Total					
Baseline	61.6 ± 15.1		56.6 ± 9.2		
Week 5	52.5 ± 15.5	<.001	47.2 ± 10.6	.003	.48
Week 9	52.7 ± 15.1	<.001	48.5 ± 8.0	.006	.21
UPDRS motor					
Baseline	27.1 ± 13.7		23.8 ± 10.6		
Week 5	22.5 ± 12.1	.009	23.7 ± 10.9	.74	.14
Week 9	24.8 ± 16.3	.025	23.8 ± 10.6	.38	.94
PDQ‐39					
Baseline	38.4 ± 25.1		23.7 ± 17.5		
Week 5	30.3 ± 25.5	.12	19.5 ± 15.2	.07	.74
Week 9	34.3 ± 25.8	.19	18.6 ± 16.0	.07	.21
ESS					
Baseline	7.5 ± 3.8		6.7 ± 4.4		
Week 5	5.8 ± 4.3	.11	5.2 ± 4.6	.13	.90
Week 9	5.5 ± 3.6	.09	5.2 ± 3.6	.17	.94
GDS					
Baseline	4.0 ± 3.3		3.3 ± 2.5		
Week 5	3.2 ± 2.7	.26	2.8 ± 2.3	.23	.71
Week 9	3.8 ± 2.8	.96	2.3 ± 2.3	.17	.09

### 3.3 Secondary outcome measures

The improvements in MFI‐General Fatigue scores were maintained at Week 9 in both groups (change in MFI‐General Fatigue scores from baseline to Week 5: acupuncture, −2.7 ± 3.6; sham, −3.5 ± 2.7; see Table 2). Again, this difference was not statistically significant between groups (ANCOVA, *p* = 0.6). Similar trends were noted in the MFI‐Total scores (change in MFI‐Total scores from baseline to Week 5: acupuncture, −8.9 ± 9.4; sham, −10.1 ± 11.2; change in MFI‐Total scores from baseline to Week 9: acupuncture, −9.7 ± 8.5; sham, −8.7 ± 10.9). The other finding of note was significant improvements in UPDRS Motor scores in the real acupuncture group compared to sham acupuncture but this difference was not statistically significant on ANCOVA.

### 3.4 Assessment of blinding

Patients were queried at Week 5 point regarding what group they thought they were in, and there were no between‐group differences in patients’ perceptions of group assignment consistent with effective blinding (chi‐square, *p* = .32).

### 3.5 Adverse events

A total of three adverse events were reported. Two were serious—one patient had a skull fracture after a fall, and the other a pelvic fracture, also after a fall. The last patient had exacerbation of anxiety. All adverse events were deemed not related to acupuncture treatment.

## 4 DISCUSSION

The results of this pilot study show that a 5‐week course of acupuncture treatment, real or sham, was effective in improving fatigue in a cohort of patients with PD. Furthermore, this improvement was maintained up to 4 weeks after completion of treatment. Apart from improved UPDRS Motor scores, real acupuncture group had no significant impact on scores of quality of life, mood, and excessive daytime sleepiness compared with sham acupuncture. In fact, there was a trend toward greater reduction in fatigue scores in the sham group (*p* = .09). To evaluate whether the improvement in fatigue is clinically meaningful, we looked at the minimal important difference of the MFI. The minimal important difference is defined as “the smallest difference in score in the domain of interest which patients perceive as beneficial and which would mandate, in the absence of troublesome side effects and excessive cost, a change in the patient's management” and is particularly useful in patient‐reported outcome measures (Jaeschke, Singer, & Guyatt, 1989). Unfortunately, no data exist on the minimal important difference of the MFI in PD. Hence, we can only extrapolate from data involving patients with rheumatological conditions, and in this group of patients, the minimal important difference for the MFI‐Total is 11.5 (Nordin, Taft, Lundgren‐Nilsson, & Dencker, 2016). Using this result, 40% of real acupuncture and 60% of sham acupuncture patients in our study had clinically meaningful improvements in fatigue.

The results are quite similar to that of a recently published randomized, controlled trial of acupuncture in fatigue in PD by Kluger et al. (2016). In their study of 94 patients, a 6‐week course of biweekly acupuncture, sham or real, resulted in significant improvements in fatigue as measured on the Modified Fatigue Impact Scale, with no significant difference between real acupuncture and sham treatment.

This was despite a major difference in study methodology on how sham acupuncture was performed. In the study by Kluger, sham acupuncture was performed using sharp round toothpicks with nonpenetration of the skin, and nonacupuncture points (defined as points 0.5 inch lateral to real acupoints) instead of real acupuncture points were “needled.” This finding appears to suggest that specific acupoint needling is not essential in the mechanism of action of acupuncture and is in accordance with previous studies (Ghaffari & Kluger, 2014).

Given that, true acupuncture is no more effective than sham acupuncture, it is likely that the effects of acupuncture on fatigue in this study are nonspecific and possibly includes a placebo response. Similar results have also been observed in trials of acupuncture in migraine, low back pain, and knee osteoarthritis (Shi, Yang, Liu, & Wang, 2012). Several factors contribute to the placebo response. These include the provider‐patient relationship, which is particularly relevant in single‐blinded studies, (Kaptchuk et al., 2008) patient expectation (Amanzio & Benedetti, 1999; Benedetti, 2013; Pollo et al., 2001) and the ritual and mystique of needling (Pollo et al., 2001). It is also suggested that complex interactions of these factors have a higher placebo effect than a pill (Kaptchuk, 2002; Kaptchuk, Goldman, Stone, & Stason, 2000; Kaptchuk et al., 2006).

This study does have some limitations. Firstly, this was a single center study and the sample size was relatively small. Secondly, in Traditional Chinese Medicine principles, key to acupuncture's therapeutic effect is the sensation of Deqi when an acupoint is needled. Patients experience Deqi as unique sensations at the needle site itself and around the site of needle manipulation, including soreness, aching, numbness, tingling, and even warmth, (Park, Park, & Lee, 2002) and a number of rating scales have been devised to measure it (Shi et al., 2012). In our study, to insure that sham needling resembled as close to real acupuncture as possible, Deqi was not mandated or measured in the real acupuncture group. This point is of relevance as some studies of acupuncture in migraine have shown that real acupuncture had statistically better outcomes than sham acupuncture when Deqi was elicited (Linde et al., 2016).

## 5 CONCLUSION

Acupuncture (real and sham) is safe and effective in reducing fatigue in a cohort of patients with PD. Given the current absence of satisfactory treatment of fatigue, there is a potential role for the use of acupuncture in the treatment of PD‐related fatigue, even if its mechanism of action is largely placebo.

## CONFLICT OF INTERESTS

None declared.
